# The Impact of Plasma Activated Water Treatment on the Phenolic Profile, Vitamins Content, Antioxidant and Enzymatic Activities of Rocket-Salad Leaves

**DOI:** 10.3390/antiox12010028

**Published:** 2022-12-23

**Authors:** Doaa Abouelenein, Ahmed M. Mustafa, Franks Kamgang Nzekoue, Giovanni Caprioli, Simone Angeloni, Silvia Tappi, Juan Manuel Castagnini, Marco Dalla Rosa, Sauro Vittori

**Affiliations:** 1 School of Pharmacy, University of Camerino, Chemistry Interdisciplinary Project (CHIP), Via Madonna delle Carceri, 62032 Camerino, Italy; 2 Department of Pharmacognosy, Faculty of Pharmacy, Zagazig University, Zagazig 44519, Egypt; 3 Department of Agricultural and Food Sciences, University of Bologna, Piazza Goidanich, 60, 47521 Cesena, Italy; 4 Interdepartmental Centre for Agri-Food Industrial Research, University of Bologna, Via Q. Bucci 336, 47521 Cesena, Italy

**Keywords:** cold plasma, polyphenols, ascorbic acid, vitamins B2, B3, antioxidant activity, peroxidase

## Abstract

Plasma activated water (PAW) recently received much attention as an alternative food preservation method. However, its effects on food quality are still scarce. This study evaluates the effect of PAW processing time on bioactive compounds of rocket-salad leaves including: 18 phenolic compounds, vitamin C, riboflavin, nicotinic acid, and nicotinamide. Moreover, the impact of PAW on both antioxidant (DPPH) and peroxidase (POD) activities was also investigated. This was performed using HPLC-DAD, HPLC-MS/MS, and spectrophotometric analysis. All treatments induced non-significant increases in total phenolic contents. However, depending on processing time, significant increases or decreases of individual phenolic compounds were observed. PAW-10 and -20 increased the ascorbic acid content to 382.76 and 363.14 mg/100 g, respectively, compared to control (337.73 mg/100 g). Riboflavin and nicotinic acid contents were increased significantly in PAW-20 (0.53 and 1.26 mg/100), compared to control (0.32 and 0.61 mg/100 g, respectively). However, nicotinamide showed non-significant increase in all treatments. Antioxidant activity improved significantly only in PAW-20, while peroxidase activity was reduced up to 36% in the longest treatment. In conclusion, PAW treatment could be an effective technique for rocket decontamination since it positively influenced the quality of rocket, improving the retention of polyphenols and vitamins.

## 1. Introduction

Rocket (*Eruca sativa* Mill.) is a vegetable medicinal plant of the Brassicaceae family which is characterized by its pleasant bitter taste. It is not only originated in the Mediterranean region, but also widely distributed all over the world [1]. Vegetables of the Brassicaceae family are generally an excellent source of different vitamins. Rocket leaves contain high levels of vitamin C together with other vitamins, such as vitamins A, K, B-1, B-2, B-3, and B-6 [2]. It also contains a large number of bioactive constituents, mainly glucosinolates and polyphenolic compounds [3]. Phenolic acids, including ferulic, coumaric, ellagic, and benzoic acids, were previously detected in rocket [4]. Regarding flavonoids, quercetin, kaempferol and isorhamnetin are the main aglycones commonly isolated from brassicaceae vegetables [5]. Moreover, the major flavonoid glycosides found in different rocket species are quercetin, kaempferol, and isorhamnetin glycosides [1,6]. Quercetin, kaempferol, and isorhamnetin aglycons together with other quercetin glycosides were previously identified and quantified by LC-ESI-MS in freeze-dried rocket leaves [3]. These compounds, due to their antioxidant properties, ensure greater protection against disorders, such as cancer and cardiovascular diseases [7].

Nowadays, one of the most important market demands is to find foods rich in bioactive compounds. To attend this demand, experts and researchers in the food industry are continuously searching for novel technologies that ensure preservation or even improving the retention of these bioactive compounds in food. However, the major post-harvesting problem of rocket vegetable is its rapid senescence, expressed primarily as yellowing and wilting [8], which is accompanied also by a loss of ascorbic acid, glucosinolates, and other bioactive compounds [9]. Therefore, it is very important to develop effective post-harvest processing methods to prolong its shelf-life as well as to preserve or even increase the content and the activity of these antioxidant compounds. This would improve the marketability of rocket and would have positive effects on its quality [2]. 

Among the known processing treatments for extending storage life of minimally processed rocket are cold storage [9], and the use of food disinfectants, such as chlorine, calcium oxide, and acetic acid [10]. However, these methods showed significant changes on the nutritional profile of rocket. Recently, other food processing technologies, such as cold atmospheric plasma, have received attention in food applications. Most studies on cold plasma are conducted by direct application over the foodstuffs to maximize the microbial inactivation effect. Few researchers have reported some negative effects like color loss and degradation of some bioactive compounds after the treatment [11]. The use of plasma activated water (PAW) has also been investigated as an alternative method for food processing in which the plasma treatment of water creates an acidic environment, resulting in changes of the conductivity and redox potential as well as the release of both reactive oxygen species and reactive nitrogen species [12]. 

However, it is fundamental to know the effect of this innovative treatment on the nutritional quality of the fresh products. Despite the extensive literature on the use of cold plasma techniques in food products, the mechanism of interaction of reactive species with the bioactive compounds is still poorly understood, both for the varieties of processing conditions related to plasma generation and to the matrix effect [13]. A decrease in the total content of phenolic compounds or specific classes with respect to treatment time was observed in white grape juice [14], blueberries [15] and fresh-cut apples [16]. On the contrary, after a short exposure to plasma reactive species, the content of certain phenolic compounds increased in apples [17] and blueberries [15] and was attributed to a physiological response of the tissue to the stress promoted by the reactive species of plasma. However, the increased exposure time enhanced the oxidative reaction and led to a gradual decrease in phenolic content and antioxidant activity. On the other hand, other authors explained that the increase in polyphenol content in fruit juices is due to the high extractability resulting from cellular structures disruption [18]. Generally, however, all authors state that the observed effect depends on the type and the stability of phenolic compounds. The effect of various processing technologies on vitamins must also be taken into account in order to maintain or even improve the nutritional properties of food products. Most studies reporting the effects of cold plasma on food products have focused on vitamin C stability [19]. However, more studies are needed to know the effects of plasma activated water technology on other vitamins present in foods [19]. 

The aim of this study is to assess the impact of PAW on the major bioactive compounds and nutritional composition of rocket-salad leaves, including phenolic composition and vitamin contents, to provide a new insight for the utilization of this novel technology as a safe alternative for rocket salad leaves decontamination. Therefore, in this work, the effect of different PAW processing times (2, 5, 10, and 20 min) on the main bioactive compounds (18 individual polyphenolic compounds and vitamins C, B2, and B3), the antioxidant activity, and the enzymatic activity of rocket leaves was studied.

## 2. Materials and Methods

### 2.1. Reagents and Standards

Quercetin and kaempferol -3-glucosides were supplied by PhytoLab (Vestenbergsgreuth, Germany), while the remaining 27 phenolic compounds analytical standards were purchased from Sigma-Aldrich (Milan, Italy). Ascorbic acid, riboflavin, nicotinamide, and nicotinic acid were purchased from Sigma-Aldrich (Milan, Italy).

Stock solution of each standard (1000 mg/L) was prepared by dissolving standards in methanol (HPLC-grade) from Sigma-Aldrich (Milano, Italy), and storing them in glass stoppered flasks at 4 °C until analysis. Working solutions of the standard were prepared at different concentrations by diluting the stock solutions with methanol. 

Ascorbic acid stock solution was prepared by dissolving 10 mg in 10 mL of 5% metaphosphoric acid (MPA) solution and stored in a glass-stoppered flask at 4 °C in the dark. Standard working solutions, at various concentrations, namely 0.5, 5, 10, 20, 50, 100, and 200 mg/L, were prepared by appropriate dilution.

Nicotinic acid and nicotinamide stock solutions were prepared by dissolving 10 mg of each standard in 10 mL of water with acetic acid (2% *v*/*v*) and stored in a glass-stoppered flasks in the dark at 4 °C. The riboflavin stock solution due to its low solubility was prepared by dissolving 10 mg in 250 mL of the same solvent. Standard working solutions were prepared at various concentrations by appropriate dilution of the stock solutions using water/acetic acid water with acetic acid (2% *v*/*v*). Formic acid (99%) was supplied from Merck (Darmstadt, Germany). Deionized water (more than 18 MΩ cm resistivity) was purified using a Milli-Q SP Reagent Water System from (Millipore, Bedford, MA, USA). All solvents used as mobile phase were filtered through a polyamide filter (0.2 μm) from Sartorius Stedim (Goettingen, Germany). A Phenex™ RC, 4-mm, 0.2-μm syringeless filter from Phenomenex (Castel Maggiore, BO, Italy) was used before injecting the samples into HPLC instruments. 

### 2.2. Plant Material

Fresh rocket leaves used in this study were obtained from the local market in Cesena (Italy). Leaves were kept in a refrigerated cell at a temperature of 2 ± 1 °C for no more than 24 h before PAW processing.

### 2.3. Corona Discharge to Produce PAW

PAW was produced using a corona discharge plasma source (AlmaPlasma s.rl., Bologna, Italy) as described in [20] using distilled water. A microsecond pulse generator (AlmaPulse, AlmaPlasma s.r.l.) is connected to a stainless-steel pin electrode, held 5 mm from the surface of 450 mL of distilled water which is stirred continuously. Operating parameters were 9 kV peak voltage and 5 kHz frequency. PAW was created by exposing distilled water to plasma for 4 min. As reported by [20], the measured concentrations of H_2_O_2_, NO_2_, and dissolved O_3_ after 4 min were 4.5 ± 0.1, 30.4 ± 0.9, and 0.3 ± 0.1 mg/L, respectively, and the pH of the PAW was 3.3. 

### 2.4. PAW Treatment

Following the water treatment, rocket samples were soaked in PAW for 2, 5, 10, and 20 min at room temperature in a product: liquid ratio of 1:20 (*w*:*v*). In an orbital agitator, materials were continually stirred during immersion. Rocket leaves were taken from PAW after dipping and wiped with absorbent paper to remove excess liquid. Two separate treatments were carried out for each treatment time. Fresh rocket leaves (untreated) were used as a control. Rocket samples from both washing replicates were promptly freeze-dried (freeze dryer model LIO2000P, 5Pascal, Milan, IT, Italy) after each treatment period, and the resulting samples were kept at −20 °C until analysis (about 2 weeks)

### 2.5. Determination of Phenolic Profile by HPLC-MS/MS

#### 2.5.1. Polyphenols Extraction 

Freeze-dried rocket powder (100 mg) was dissolved in 10 mL of aqueous ethanol 70% acidified with 1.5% formic acid. The suspension was shaken vigorously for 3 min and then put in the sonicator using a FALC ultrasonic bath (FALC, Treviglio, Italy) for 60 min at a frequency of 40 kHz, and a temperature of 30 °C. After extraction, centrifugation for 10 min at 5000 rpm was performed. The clear supernatant was filtered through a 0.2-μm syringeless filter before injection. Triplicate analysis was performed for each sample, and results were used for both HPLC-MS/MS analysis and DPPH reducing activity determination 

#### 2.5.2. HPLC-MS/MS Analysis

The phenolic compounds in the rocket extracts were analyzed using the method proposed by [21] with some modifications. Agilent 1290 Infinity series was used for performing HPLC analysis. The series was coupled with Triple Quadrupole 6420 purchased from Agilent Technology (Santa Clara, CA, USA). The operation was done in both negative and positive electrospray ionization (ESI) modes. The phenolic compounds were separated on a Synergi Polar–RP C18 analytical column (250 mm × 4.6 mm, 4 µm) from Phenomenex (Chesire, UK), using a mixture of water as solvent A and methanol as solvent B, both with formic acid 0.1%, in gradient elution mode. The following elution program was used for separation: isocratic condition from 0 to 1 min, followed by 20% mobile phase B from 1 to 25 min, 20–85% mobile phase B from 25 to 26 min, then an isocratic condition was used for 85% mobile phase B. Finally, from 26 to 32 min, from 85% to 20% mobile phase B was used. The injection volume of samples was 2 μL, and the flow rate was set at 0.2 mL/min. Dynamic-multiple reaction monitoring mode was used for detecting compounds, and the peak areas were integrated for quantification. The mass spectrometer parameters for the analyzed compounds are reported in Table 1.

### 2.6. Determination of Ascorbic Acid Content Using HPLC-DAD

#### 2.6.1. Ascorbic Acid Extraction

The content of ascorbic acid was determined by an HPLC-DAD method previously described by [22]. Hence, 100 mg freeze-dried rocket samples were immersed in a 5 mL extraction solution of water containing 5% MPA. The extraction was performed or 4 h in darkness using a magnetic stirrer. Then, the samples were centrifuged for 10 min at 5000 rpm. The clear supernatant was filtered using a 0.45-mm membrane filter before analysis.

#### 2.6.2. HPLC-DAD Analysis

Hewlett Packard (Palo Alto, CA, USA) HP-1090 Series II was used for HPLC analysis. The series is composed of an autosampler and a binary solvent pump, together with a diode-array detector (DAD). Water with 0.1% formic acid (90%) was utilized as mobile phase A and methanol with 0.1% formic acid (10%) was used as mobile phase B. The used flow rate was 0.5 mL/min in isocratic conditions. The injection volume was 10 µL. The used analytical column was a Synergi Polar-RP C18 (4.6 mm × 150 mm, 4 µm) from Phenomenex (Chesire, UK). UV spectra were recorded (from 210 to 400 nm), and 245 nm was used for quantification. Results were expressed as mg/100 dry weight (DW).

### 2.7. Simultaneous Quantification of Riboflavin, Nicotinamide and Nicotinic Acid by UHPLC–MS/MS 

#### 2.7.1. Acidic Hydrolysis Extraction of Riboflavin, Nicotinamide and Nicotinic Acid 

The content of riboflavin (vitamin B2), nicotinamide, and nicotinic acid in rocket samples was detected. The analysis was carried out using the method of [23]. 0.1 N hydrochloric acid (10 mL) was used to hydrolyze 200 mg of each sample for 30 min at 100 °C with magnetic stirring. The liquid was allowed to cool to 25 °C before being adjusted to a pH of 4.0–4.5 using 2 M sodium acetate. After that, a 1 mL sample was diluted to 5 mL with deionized water before being filtered through Whatman No. 40 filter paper. Before injection, the supernatant was filtered again using a 0.2 m membrane filter (Gelman Sciences, Ann Arbor, MI, USA).

#### 2.7.2. UHPLC-ESI–MS/MS Analysis

A previous approach was used to measure riboflavin, nicotinic acid, and nicotinamide levels [23]. An Agilent 1290 Infinity series and a Triple Quadrupole 6420 from Agilent Technology (Santa Clara, CA, USA) were used to perform the analysis, which used an electrospray (ESI) source in the positive ionization mode. A Kinetex Hilic analytical column (100 mm 4.6 mm i.d., particle size 2.6 m) from Phenomenex was used to separate B vitamins (riboflavin, nicotinic acid, and nicotinamide) (Torrance, CA, USA). For UHPLC–MS/MS analysis, the mobile phase was a combination of water (A, 95%) and acetonitrile (B, 5%), both containing formic acid 0.1%, and flowed at 0.8 mL/min with isocratic elution. The injection was 2 μL in volume. The column temperature was 30 °C, while that of the drying gas in the ionization source was 300 °C. The gas flow rate was set to 12 L/min, the nebulizer pressure was set to 50 psi, and the capillary voltage was set to 4000 V. The data were collected in multiple reaction monitoring mode, with the most abundant transitions chosen for quantification and the others for qualifying. The results were given in mg per 100 g of dry weight (DW).

The mobile phase used for analysis was a mixture of 95% water with 0.1% formic acid (mobile phase A) and 5% acetonitrile with formic acid 0.1% (mobile phase B). Isocratic elution was done at a flow rate of 0.8 mL/min. The samples injection volume was 2 μL and the gas flow was set at 12 L/min. The temperature of the column was kept at 30 °C while the temperature of the drying gas in the ionization source was kept at 300 °C. The acquisition was performed in multiple reaction monitoring mode and the most abundant transitions were used for quantitation. Results were expressed as mg per 100 DW.

### 2.8. DPPH Reducing Activity Determination

The antioxidant activity was determined using the DPPH method according to [24]. In which 100 mg lyophilized rocket powder were extracted with 10 mL of 70% aqueous ethanol acidified with 1.5% formic acid. Then, 0.5 mL of this extract was mixed with 4.5 mL of DPPH solution. The mixture was shaken and then kept in darkness for 30 min. The absorbance was measured at 517 nm. The used reference antioxidant was Trolox. Results were expressed as mg trolox equivalent (TE)/100 g dry weight (DW). 

### 2.9. Peroxidase (POD) Activity

POD activity was assayed using the spectrophotometric method described by Tappi, et al. [25]. Hence, 5 g of sample were mixed for 2 min with 50 mL of 0.1 M potassium phosphate buffer (pH 6.5) with a T25 digital Ultraturrax (IKA^®^-Werke GmbH & Co. KG, Staufen, Germany). After filtration, the solution was centrifuged for 10 min at 4 °C and 10,000× *g*, and the supernatant was collected and considered as enzymatic extract. 

A solution containing 99.8 mL of 0.1 M potassium phosphate buffer (pH 6.5), 0.1 mL of 99.5% guaiacol and 0.1 mL of 30% hydrogen peroxide was considered as POD substrate. Enzymatic activity was assessed mixing 150 μL of enzymatic extract to 3 mL of substrate solution in 10-mm pathlength glass cuvettes followed by monitoring the increase in absorbance at 470 nm at 25 °C for 3 min. 

Results were expressed as residual enzymatic activity (%) considered as the ratio between PAW treated sample versus untreated one and measured on three independent extracts.

### 2.10. Statistical Analysis

Measurements were performed in triplicate. A one-way analysis of variance (ANOVA) was used for evaluation. Tukey’s test with 95% confidence level was applied. Analysis was performed using Minitab ver. 19.0 and Microsoft Excel 365.

## 3. Results and Discussion

### 3.1. Phenolic Profile of Rocket Leaves

Before the analysis of different rocket extracts, the HPLC-MS/MS analytical method was validated by evaluating some method-performance parameters such as linearity, sensitivity, limits of detection (LODs), limits of quantification (LOQs), and precision. Evaluation of the linearity was done by injecting seven different known concentrations from 0.005 to 10 μg/mL of the 29 standard analytes that showed good linearity (R^2^ ≥ 0.9943). The calculation of LODs and LOQs was conducted by injecting serial dilutions of the standard solutions, taking the signal-to-noise ratio of 3:1 for the LOD and 10:1 for the LOQ, respectively. The range of LODs was ranged from 0.0004 to 0.0033 mg/L, while the LOQs range was defined in the range 0.0012 to 0.01 mg/L. Intraday precision was validated by injecting the standard mix solution five times per day, while interday precision measurements were performed for three consecutive days once per day.

A very good precision of the method was revealed with interday variations and intraday variations as relative standard deviations (RSDs %) ranged from 0.34% to 4.73% and from 0.12% to 2.62%; respectively. The method showed high specificity and the stability of the retention time for each analyte was detected three times over a period of three days where RSDs% in all cases was less than or equal to 1.0% [21]. 

**Figure 1:**
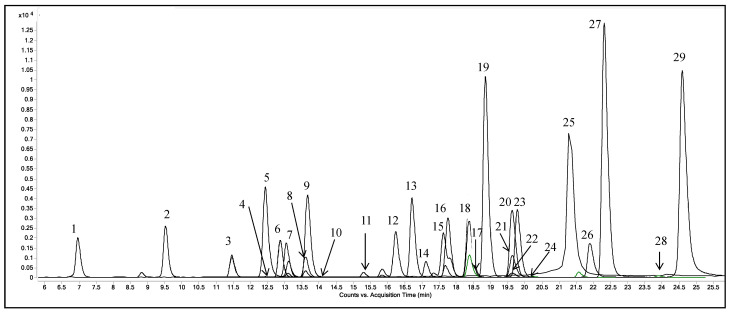
HPLC-MS/MS chromatogram of a standard mixture of 29 phenolic compounds plotted as overlapped “dynamic-Multiple Reaction Monitoring” (MRM) transition of each analyte. (1) Gallic acid, (2) Neochlorogenic acid, (3) (+)-Catechin, (4) Procyanidin B2, (5) Chlorogenic acid, (6) *p*-Hydroxybenzoic acid, (7) (−)-Epicatechin, (8) 3-Hydroxybenzoic acid, (9) Caffeic acid, (10) Vanillic acid, (11) Syringic acid, (12) Procyanidin A2, (13) P Coumaric acid, (14) Ferulic acid, (15) 3,5-Dicaffeoylquinic acid, (16) Rutin, (17) Hyperoside, (18) Isoquercitrin, (19) Phloridzin, (20) Quercitrin, (21) Myricetin, (22) Naringin, (23) Kaempferol-3-glucoside, (24) Hesperidin, (25) Ellagic acid, (26) Quercetin, (27) Phloretin, (28) Kaempferol, (29) Isorhamnetin.

Figure 1 shows the chromatogram of the 29 analytes standard mixture shown as overlapped dynamic-MRM transition for each detected analyte. A total of 18 phenolic compounds were detected in control and PAW treated rocket samples. The contents of individual and total phenolic compounds are given in Table 2. 

Since high temperatures used usually in food processing are able to degrade polyphenols, non-thermal technologies (especially, cold plasma technology) have become one of the best alternatives for an increased shelf life with improved polyphenol retention [26]. Therefore, the phenolic profiles of control and PAW-treated rocket samples were analyzed by HPLC-MS/MS (Table 2). The phenolic profile of the control rocket sample consisted mainly of quercetin and quercetin glycosides (around 57% of the total), which is consistent with the previous study of [27] who reported that quercetin derived compounds were the major flavonoids in rocket samples. However, previous studies have shown variability in rocket’s phenolic profile that could be due to several factors, including extraction, chromatographic method together with quantification tools, as well as the origin and condition of the sample [6]. In the current study, the extracts of the rocket leaves treated by PAW for 2, 5, 10, and 20 min were compared with the control. The treatment time was found to promote a general increase in the total phenolic content of rocket for all treatment times with a maximum increase of 27.1% in the samples treated with PAW-20. However, this increase in total phenolic content was not statistically significant. On the other side, analyzing the singular content of the different compounds, various significant differences were found, mostly at the longer treatment times. 

Among the phenolic acids, the extracts of rocket samples treated by PAW-20 min showed significant increase in ferulic, caffeic and coumaric acids concentrations compared to the control. Moreover, a significant increase in the concentration of chlorogenic acid was observed, but only in samples treated for 2 and 5 min. However, the concentration of 3,5-dicaffeoylquinic acid (isochlorogenic acid) was significantly decreased after PAW treatment, while ellagic acid concentration was constant in all treated samples. An increase in hydroxycinnamic acids has been observed by various authors after cold plasma exposure. Herceg et al. [28] reported a higher content of chlorogenic, ellagic, and ferulic acid together with an increase in the catechin content of pomegranate juice processed by gas phase plasma. The authors attributed this increase to an effect of cell membrane breakdown, hydrolysis, and depolymerization due to the reactive species of plasma. Moreover, a 30% increase in the content of chlorogenic acids was observed after 10 min treatment in fresh-cut apples [17]. However, in this case, authors suggested an increase in the gene expression of enzymes involved in the biosynthesis of phenolic compounds from the phenylpropanoid pathway, including: cinnamate-4-hydroxylase, 4-coumarate coenzyme A ligase, and phenylalanine ammonium lyase enzymes, as it occurs after wounding of vegetable tissue. The exposure to highly reactive species generated by plasma discharges in PAW represents a physiological stress for the tissue and such species can act as abiotic elicitors participating in the regulation of stress responses in plants, which explains the stimulating effect of plasma treatments on polyphenol biosynthesis in plant tissues [29]. 

Compared to the control, the extracts of rocket samples treated by PAW-20 showed a significant increase in quercetin, quercetin-3-O-galactoside, kaempferol-3-O-glucoside and hesperidin concentrations (79.8%, 58.6%, 113.0%, and 33.3%, respectively). A non-significant increase in the concentration of quercetin-3-O-glucoside was found with PAW-treated samples. On the contrary, a reduction of the concentrations of quercetins-3-O-rutinoside and -3-O-rhamnoside was observed after 5 min of processing time for both compounds. Similarly, a significant increase in quercetin concentration was reported in grape pomace treated by high voltage cold atmospheric plasma in samples treated for 15 min [30]. Moreover, an increase in the content of both quercetin and quercetin glycoside after 10 min treatment of fresh-cut apples was reported by [17], which is suggested to be a result of the cell wall hydrolysis [31]. On the other hand, a reduction in the concentration of quercetin and kaempferol glycosides was found in *Pisum sativum* treated by cold atmospheric plasma [32].

The content of dihydrochalcone derivative phlorizin showed a significant increase (25%) after 10 min (PAW-10), followed by a significant reduction in PAW-20 samples. Similarly, a 40% increase in phlorizin concentration was previously reported after 10 min treatment with cold plasma in fresh cut apples, which was suggested to be synthesized after the increase in the activity of the enzymes involved in the phenyl-propanoid biosynthetic pathway like chalcone synthase and phenylalanine ammonia lyase enzymes [17]. On the other hand, a significant decrease in phloretin levels was observed only 2 min of plasma activated water treatment and remained unchanged over a longer treatment period. In general, it can be assumed that reactive species of the plasma can interact with the surface of the food, causing a physiological response that converts these reactive particles into compounds that are less harmful to plant cells [33]. In addition, this response was associated by the significant increase in the activity and genes expression of some enzymes responsible for polyphenol preservation [29]. Some authors also reported that after a stimulation of the phenolic compounds production, increasing treatment time led to a following reduction, attributed to oxidative or polymerization reactions [16,34]. However, this effect was observed for prolonged exposition times (more than 40 min) which were not reached in the present study.

Nevertheless, it is important to consider that most of the previous literature findings are based on the exposure of fruit and vegetable tissues to gas phase plasma. Hence, while both plasma phases contain highly reactive species, their specific composition may vary quite substantially.

### 3.2. Ascorbic Acid Content

**Figure 2:**
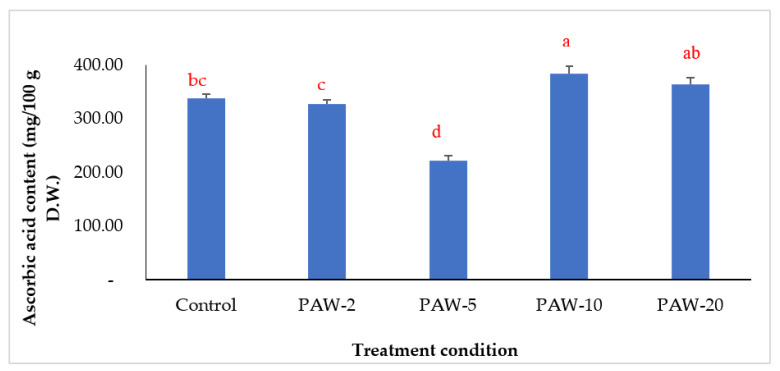
Changes in ascorbic acid content of PAW treated rocket samples at different treating times compared to control samples. Means that do not share letters differ significantly (*p* < 0.05) according to Tukey’s test. Legends: PAW-2, PAW-5, PAW-10, and PAW-20 refer to rocket samples subjected to plasma-activated water (PAW) treatment for 2, 5, 10, and 20 min, respectively.

Vitamin C has high antioxidant capacity which inactivates free radicals and other reactive substances that cause oxidative damage to biomolecules such as proteins, lipids and DNA [35]. Researchers use vitamin C as an indicator of food quality because it is a very sensitive bioactive compound that provides a valid mark for the loss of vitamins and other nutritional components [36]. The contents of ascorbic acid of the non-treated and PAW treated samples are shown in Figure 2. It can be observed that treatment time has a significant influence (*p* < 0.05) on the content of ascorbic acid. The highest vitamin C concentration (382.76 mg/100 g DW) was found in the product subjected to intermediate processing time (10 min). Longer treatment (20 min) resulted in a non-significant increase of vitamin C content (363.14 mg/100 g), compared with control sample (337.73 mg/100 g D.W.). Contradictory reports were found in the literature regarding the effect of cold plasma processing on the contents of vitamin C in vegetables. A decrease in the content of ascorbate in the roots of tomato seedlings was observed in PAW-15 and PAW-30 samples [37] and in orange juice [38]. However, the ascorbic acid contents of blueberry samples and guava-flavored whey beverages were significantly enhanced post plasma treatment [15,39]. 

Reactive plasma particles, such as nitric oxide, can modulate the ascorbate-glutathione cycle and enhance the activity of the dehydroascorbate reductase enzyme that converts dehydroascorbic acid (the oxidized form of ascorbic acid) to ascorbic acid. It is therefore possible that, under mild conditions, the regeneration of ascorbic acid is greater than its reduction by the action of the reactive species of plasma [39]. However, according to these authors, the increase of treatment time and rate of flow led to a reduction of ascorbic acid levels.

Finally, vitamin C content was only slightly influenced by plasma exposure in radish sprouts [40], kiwifruit [34], and various minimally processed vegetables. Generally, the overall effect on vitamin C observed in the present study is not negative.

### 3.3. Vitamins B (Riboflavin, Nicotinamide, and Nicotinic Acid) Contents

In this study, the contents of different forms of vitamin B were evaluated using the UHPLC-ESI–MS/MS system. The contents of the untreated (control) and the PAW treated rocket samples are shown in Table 3. Compared to the control sample (0.32 mg/100 g), PAW-2, PAW-5, and PAW-10 exhibited non-significant changes of riboflavin (B2) content. On the contrary, PAW-20 sample showed a significant increase in riboflavin content with a value of 0.53 mg/100 g (Table 3). Since riboflavin has a pH dependent stability, being more stable under acidic conditions [41], the acidic environment provided by PAW- treatment may be responsible for the retention of this vitamin after treatment. 

Vitamin B3 is composed by two main forms, niacin, or nicotinic acid, and nicotinamide, that have the same vitaminic activity. A non-significant change (*p* > 0.05) was observed in the nicotinamide content of all PAW treated samples, while a significant enhancement of nicotinic acid content was observed with PAW-20 (1.26 mg/100 g D.W.), compared with control sample (0.61 mg/100 g D.W). Previous reports indicated that an increase in the concentration of niacin after food thermal processing may be due to the change of bound niacin to the free form (nicotinic acid and nicotinamide) [42]. However, as PAW is a non-thermal treatment, this effect might be considered negligible. Moreover, to our knowledge, the production of newly formed riboflavin and nicotinic acid has never been observed as a consequence to metabolic stress. Therefore, the observed increase in the longer processing time could be related to a higher extraction capacity due to the alteration of the integrity of the cell membrane promoted by the reactive species of the plasma [17]. 

### 3.4. DPPH Reducing Activity 

**Figure 3:**
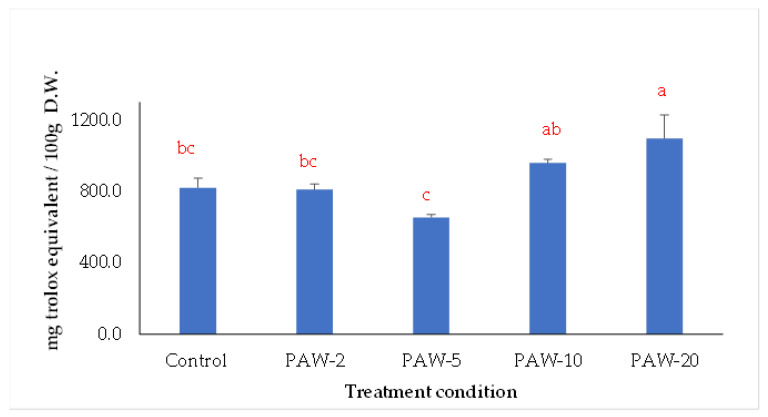
DPPH reducing activity expressed as mg trolox equivalent (TE)/ 100 g dry weight (D.W.) of plant material of PAW- treated rocket samples at different treating times compared to control samples. Means that do not share letters differ significantly (*p* < 0.05) according to Tukey’s test.PAW-2, PAW-5, PAW-10, and PAW-20 refer to rocket samples subjected to plasma-activated water (PAW) treatment for 2, 5, 10, and 20 min, respectively.

The major antioxidant compounds in fruits and vegetables are vitamins (C and E) together with the phenolic compounds. These bioactive compounds are able to scavenge free radicals, which are the cause of many diseases affected by oxidative stress. Thus, changes in DPPH reducing activity are thought to be associated with the changes in these compounds. Here, PAW treatment affected the DPPH reducing activity of the rocket leaves. The DPPH reducing activity was increased in PAW-20 and PAW-10 samples and decreased in PAW-5 and PAW-2 samples. PAW-20 showed the highest DPPH reducing activity, while PAW-5 demonstrated the lowest activity. As shown in Figure 3, a reduction in the DPPH reducing activity (20.36%), measured as radical scavenging ability, was observed in the PAW-5 sample, which might be due to the observed decrease in the ascorbic acid content (by 35.56%), which has been proven to have strong antioxidant properties. On the other side, a significant increase compared to the untreated sample was observed for the PAW-20 sample, reflecting what was observed for the polyphenol and partly the vitamin content. In polyphenols, the anti-free radical activity depends on the specific structure (presence of phenolic hydrogens) and on the stabilizing capacity of the resulting phenoxy radicals. Generally, while increasing the hydroxyl groups and decreasing the glycosylation led to higher antioxidant activity [43], it is quite difficult to precisely predict the antioxidant capacity of a mixture of polyphenols. Previous reports on button mushroom showed an increase in the antioxidant activity with increase in PAW treatment time with the best antioxidant activity with PAW-15 [44]. Although there are few data on the effect of PAW treatment on the functional and nutritional characteristics of foods [13], antioxidant activities generally follow a similar trend to the bioactive compounds. 

### 3.5. POD Activity

**Figure 4:**
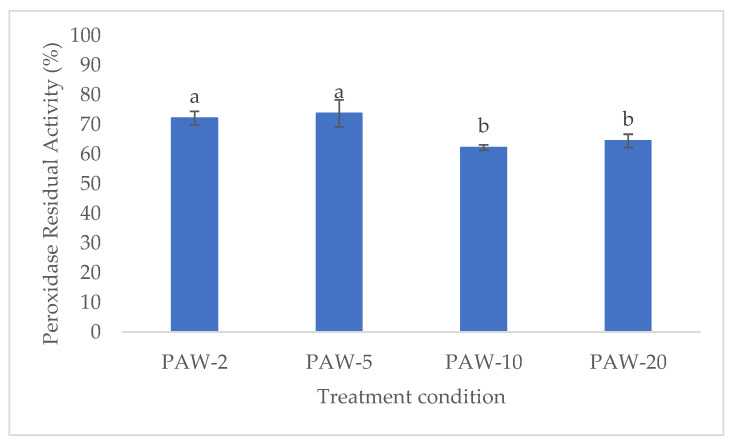
Peroxidase Residual Activity (RA) (%) in PAW treated rocket samples processed at different times. Means that do not share letters differ significantly (*p* < 0.05).

POD is an oxidative enzyme, found ubiquitously in vegetable tissues and can cause quality degradation through oxidation of various substrates, such as phenolic compounds, lipids, and pigments [45]. As shown in Figure 4, PAW treatment allowed a significant reduction of the POD activity compared to the untreated sample. Residual activity was around 73% after 2- and 5-min treatments and decreased to around 63% when dipping was prolonged to 10 and 20 min. 

The inactivation of enzymes following plasma exposure is being observed by a variety of researchers in both model systems [46,47,48] and real food products [25,49], and the accepted mechanism is the oxidation of the reactive side chain of the amino acid residues, leading to a modification of the secondary structure and loss of functionality. The inactivation of degradative enzymes in vegetable tissue represents a very positive effect of plasma application, as it reduces the rate of quality depletion during storage, favoring the increase in shelf-life. 

The majority of studies on enzymatic inactivation have been carried out after exposure to gaseous plasma, while the effect of PAW is less investigated. However, Xu et al. [45] observed a reduction of enzymatic browning during storage of button mushrooms immersed in PAW up to 15 min. Although they did not directly measure the activity of the enzyme, the authors attributed the effect to the inactivation of polyphenoloxidase (PPO).

## 4. Conclusions

This is the first study showing the effect of PAW technique on the phytochemical profile, nutritional properties, antioxidant, and enzymatic activity of rocket leaves. From the obtained results, it is possible to conclude that the PAW treatment at longer treatment times allowed a good preservation of the investigated vitamins (ascorbic acid, riboflavin, nicotinic acid, and nicotinamide) and polyphenolic compounds. The longer treatment (20 min) actually promoted an increase of the content of vitamins B2 and B3 as well as some selected polyphenols. This result might be due to a combined effect of membrane damage favoring component extraction as well as the physiological response promoting the endogenous production of secondary metabolites. Moreover, the DPPH reducing activity of the treated samples showed the same trend of the content of bioactive compounds, showing a significant increase in the longest treatment. A significant reduction of peroxidase activity was observed, which might lead to higher quality preservation during storage. However, it is important to highlight the need for further analysis to better understand the mechanisms behind some of the observed effects and to assess the antioxidant activity with in-vivo methods. In conclusion, the PAW showed good and positive impacts on the studied compounds of rocket, which is very important regarding its acceptance as an alternative food processing technique. 

## Figures and Tables

**Table 1:** HPLC–MS/MS acquisition parameters (dynamic-MRM mode) used for the analysis of the 29 phenolic compounds.

No.	Compounds	Precursor ion, *m*/*z*	Production, *m*/*z*	Fragm-entor, V	Collision Energy, V	Polarity	Retention Time (Rt, min)
1	Gallic acid	169	125.2 *	97	12	Negative	6.96
2	Neochlorogenic acid	353	191.2 *, 179	82	12, 12	Negative	9.52
3	(+)-Catechin	289	245.2 *,109.2	131	8, 20	Negative	11.44
4	Procyanidin B2	576.99	576.99 *, 321.2	160	0, 32	Negative	12.41
5	Chlorogenic acid	353	191.2 *, 127.5	82	12, 20	Negative	12.42
6	p-Hydroxybenzoic acid	137	93.2 *	92	16	Negative	12.86
7	(-)-Epicatechin	289	245.1 *, 109.1	126	8, 20	Negative	13.03
8	3-Hydroxybenzoic acid	137	93.2 *	88	8	Negative	13.59
9	Caffeic acid	179	135.2 *, 134.1	92	12, 24	Negative	13.65
10	Vanillic acid	167	152.4 *, 108.1	88	12, 20	Negative	14.32
11	Syringic acid	196.9	182.2 *, 121.2	93	8, 12	Negative	15.28
12	Procyanidin A2	575	575 *, 285	170	0, 20	Negative	16.18
13	P-Coumaric acid	163	119.2 *, 93.2	83	12, 36	Negative	16.70
14	Ferulic acid	193	134.2 *, 131.6	83	12, 8	Negative	17.10
15	3,5-Dicaffeoylquinic acid	514.9	353.1 *, 191	117	8, 28	Negative	17.61
16	Rutin	609	300.2 *, 271.2	170	32, 50	Negative	17.73
17	Hyperoside	465.01	303 *, 61.1	97	8, 50	Positive	18.33
18	Isoquercitrin	463	271.2 *, 300.2	155	44, 24	Negative	18.36
19	Phloridzin	435.39	273 *, 167	155	8, 28	Negative	18.83
20	Quercitrin	446.99	300.2 *, 301.2	160	24, 16	Negative	19.61
21	Myricetin	316.99	179.1 *, 182	150	16, 24	Negative	19.61
22	Naringin	578.99	271.3 *, 151.3	170	32, 44	Negative	19.62
23	Kaempferol-3-glucoside	447	284.2 *, 255.2	170	24, 40	Negative	19.77
24	Hesperidin	611.01	303 *, 334.8	112	20, 12	Positive	20.19
25	Ellagic acid	301	301 *, 229	170	0, 24	Negative	21.41
26	Quercetin	300.99	151.2 *, 179.2	145	16, 12	Negative	21.87
27	Phloretin	272.99	167 *, 123	116	8, 20	Negative	22.30
28	Kaempferol	287.01	153 *, 69.1	60	36, 50	Positive	23.84
29	Isorhamnetin	314.99	300.2 *, 196.1	145	16, 4	Negative	24.57

* These product ions were used for quantification.

**Table 2:** Content of phenolic compounds (mg kg^−1^, D.W.) determined by HPLC-MS/MS in control and PAW treated rocket samples processed at different times.

Class	Compound	Treatment Condition (Time)				
		Control	PAW-2	PAW-5	PAW-10	PAW-20
	Ellagic acid	26.2 ± 7.8 a	32.0 ± 1.6 a	23.0 ± 5.2 a	27.2 ± 4.6 a	23.6 ± 2.5 a
Phenolic acids	Chlorogenic acid	4.9 ± 0.7 a	7.7 ± 0.5 b	5.6 ± 0.1 a	9.4 ± 0.4 c	4.8 ± 0.1 a
	3,5-Dicaffeoylquinic acid	3.6 ± 0.2 a	2.0 ± 0.2 b	1.1 ± 0.2 c	1.1 ± 0.2 c	0.8 ± 0.2 c
	4-Hydroxy benzoic acid	1.9 ± 0.0 a	1.0 ± 0.0 b	1.2 ± 0.0 b	1.7 ± 0.1 a	1.7 ± 0.2 a
	Ferulic acid	1.7 ± 0.2 a	1.5 ± 0.2 a	1.9 ± 0.2 a	1.4 ± 0.2 a	3.8 ± 0.3 b
	Caffeic acid	0.4 ± 0.0 a	0.3 ± 0.0 a	0.3 ± 0.0 a	0.3 ± 0.0 a	0.9 ± 0.1 b
	P- Coumaric acid	0.3 ± 0.0 a	0.3 ± 0.1 a	0.6 ± 0.1 a	0.6 ± 0.1 a	0.8 ± 0.0 b
Flavonol aglycons	Quercetin	11.4 ± 3.4 a	9.3 ± 2.1 a	16.6 ± 2.7 ab	9.9 ± 0.6 a	20.5 ± 0.9 b
	Isorhamnetin	10.5 ± 0.9 ab	6.3 ± 0.9 bc	13.5 ± 1.0 a	3.7 ± 0.0 c	9.5 ± 1.8 ab
	Kaempferol	0.7 ± 0.1 ab	0.4 ± 0.0 a	1.2 ± 0.2 c	0.4 ± 0.0 a	1.0 ± 0.1 bc
Flavonol glycosides	Quercetin-3-O-galactoside	37.0 ± 2.9 a	43.0 ± 0.8 ab	52.5 ± 0.4 ab	50.6 ± 0.5 ab	58.7 ± 6.7 b
	Quercetin -3-O-glucoside	26.4 ± 4.7 a	32.2 ± 0.3 a	32.7 ± 6.0 a	31.9 ± 1.4 a	33.6 ± 5.7 a
	Quercetin-3-O-rutinoside	2.7 ± 0.0 a	2.5 ± 0.2 a	1.0 ± 0.2 b	0.6 ± 0.0 b	0.9 ± 0.0 b
	Quercetin-3-O-rhamnoside	1.8 ± 0.3 a	1.9 ± 0.2 a	1.0 ± 0.2 b	0.8 ± 0.2 b	0.7 ± 0.0 b
	Kaempferol-3-O-glucoside	4.6 ± 0.1 a	5.1 ± 0.7 ab	9.3 ± 2.3 bc	6.2 ± 0.3 abc	9.8 ± 0.5 c
Flavanone glycoside	Hesperidin	3.0 ± 0.3 ab	2.3 ± 0.1 a	4.9 ± 1.3 b	3.8 ± 0.1 ab	4.0 ± 0.1 ab
Dihydro- chalcones	Phloretin	0.3 ± 0.0 a	0.1 ± 0.0 b	0.1 ± 0.0 b	0.1 ± 0.0 b	0.1 ± 0.0 b
	Phlorizin	1.6 ± 0.0 a	1.8 ± 0.0 ab	1.9 ± 0.1 b	2.0 ± 0.1 b	1.2 ± 0.1 c
Total phenolics		138.8 ± 13.3 a	149.7 ± 1.8 a	168.5 ± 9.6 a	151.7 ± 10.3 a	176.4 ± 18.1 a

All the data are expressed as mean ± standard deviations. Means that do not share letters in each row differ significantly (*p* < 0.05) according to Tukey’s test. Legends: PAW-2, PAW-5, PAW-10, and PAW-20 refer to rocket samples subjected to plasma-activated water (PAW) treatment for 2, 5, 10, and 20 min, respectively.

**Table 3:** Riboflavin, nicotinic acid and nicotinamide contents of control and PAW treated rocket samples expressed in (mg/100 g).

Treatment Conditions	Riboflavin	Nicotinic Acid	Nicotinamide
Control	0.32 ± 0.00 ^bc^	0.61 ± 0.01 ^bc^	1.27 ± 0.22 ^a^
PAW-2	0.23 ± 0.01 ^c^	0.46 ± 0.04 ^d^	1.41 ± 0.16 ^a^
PAW-5	0.40 ± 0.03 ^ab^	0.65 ± 0.06 ^b^	1.43 ± 0.12 ^a^
PAW-10	0.32 ± 0.06 ^bc^	0.48 ± 0.02 ^cd^	1.64 ± 0.17 ^a^
PAW-20	0.53 ± 0.02 ^a^	1.26 ± 0.02 ^a^	1.63 ± 0.02 ^a^

All the data are expressed as mean ± standard deviations. Means that do not share superscript letters in each column differ significantly (*p* < 0.05) according to Tukey’s test with *p* < 0.05. Note: PAW-2, PAW-5, PAW-10, and PAW-20 refer to plasma-activated water (PAW) subjected to plasma activated water treatment for 2, 5, 10, and 20 min respectively.

## Data Availability

The data presented in this study are available in the article.
